# Evaluation of the Effect of Surgery Type on Preoperative Anxiety in Oral and Maxillofacial Surgery Patients

**DOI:** 10.1155/ijod/4149619

**Published:** 2026-05-13

**Authors:** Zeynep Busra Gur, Ozlem Kocaturk, Burcu Gursoytrak

**Affiliations:** ^1^ Department of Oral and Maxillofacial Surgery, Faculty of Dentistry, Aydin Adnan Menderes University, Aydın, Türkiye, adu.edu.tr; ^2^ Divison of Anesthesiology, Department of Oral and Maxillofacial Surgery, Faculty of Dentistry, Aydin Adnan Menderes University, Aydın, Türkiye, adu.edu.tr

**Keywords:** pain, postoperative nausea and vomiting, preoperative anxiety, recovery period, surgery procedures

## Abstract

**Introduction:**

In most surgical operations, the type of surgery has been shown to influence preoperative anxiety, which in turn leads to changes during surgery. The aim of this study is to investigate how preoperative anxiety is affected by the type of surgery in patients undergoing maxillofacial surgery under general anesthesia.

**Material and Methods:**

This prospective comparative observational study involved patients who underwent oral and maxillofacial surgery at Aydın Adnan Menderes University. Patients were divided into the minor surgery and the major surgery groups. Preoperative anxiety measured by the Spielberger State–Trait Anxiety Inventory‐State (STAI‐S) and STAI‐Trait (STAI‐T) and the Amsterdam Preoperative Anxiety and Information Scale (APAIS). Postoperative pain measured by visual analog scale (VAS) at the first and fourth hours, postoperative nausea and vomiting (PONV) were observed within 6 h.

**Results:**

A total of 120 patients were included in the study; 60 in the major surgery group and 60 in the minor surgery group. Preoperative anxiety scores were significantly higher in the minor surgery group compared to the major surgery group (STAI‐S: 49.15 ± 12.82 vs. 43.26 ± 13.70; STAI‐T: 45.65 ± 10.97 vs. 39.90 ± 7.48; *p* = 0.017 and *p* = 0.001, respectively). Patients in the major surgery group had significantly higher pain scores at 1 h postoperatively, higher frequency of vomiting, and longer recovery times compared to patients in the minor surgery group. (*p*  < 0.05).

**Conclusion:**

Preoperative anxiety can affect patients in the intraoperative and postoperative periods. This study, which evaluated the effect of surgery type on anxiety, found that patients who underwent minor surgery showed higher anxiety scores compared to patients who underwent major surgery. Since there is a multifactorial relationship between pain, postoperative discomfort, and preoperative anxiety; this study found that the type of surgery the patient underwent was a more influential factor than anxiety in terms of pain and postoperative discomfort.

## 1. Introduction

Anxiety is defined as restlessness, worry, fear, and tension and is a response to internal and external stimulants through emotional, behavioral, and physical symptoms [1]. Preoperative anxiety affects patients during and after surgery, increasing anesthetic drug consumption, delaying wound healing, and prolonging hospital stays [2, 3]. Hence, investigating the causes of preoperative anxiety is important to develop effective treatment methods. Many studies have shown that preoperative anxiety is associated with various factors, including the patient’s age, gender, education level, type of surgery, and experience [1, 4, 5]. In many surgical procedures, the type of surgery has been shown to affect preoperative anxiety and lead to changes during surgery [6–8]. To the best of our knowledge, there are limited studies in the maxillofacial surgery literature investigating how the type of surgery affects preoperative anxiety. Identifying risk factors associated with anxiety in patients undergoing oral and maxillofacial surgery can guide the provision of the best possible preoperative and postoperative care. The primary objectives of this study were as follows:

1. To compare preoperative anxiety levels between patients undergoing minor and major oral and maxillofacial surgery under general anesthesia.
2. To compare postoperative pain and recovery outcomes between these groups.

## 2. Materials and Methods

### 2.1. Patients

The prospective comparative observational study included patients who would be hospitalized in the Surgery Inpatient Service of the Faculty of Dentistry, who could communicate, were literate in Turkish, were in the age range of 18–65 years, were in ASA I–II groups, and would undergo oral and maxillofacial surgery. The total number of patients to be included in the study was determined to be 60 by calculating the sample size [9]. Patients who had a neurological or psychological disorder, had a history of psychiatric drug use, were administered anxiolytic or sedative medicine before the surgery, needed anxiolytic and opioid drugs in the postoperative period, and were operated on outside the routine surgery and anesthesia procedure were excluded from the study. After written consent forms were obtained from the patients, face‐to‐face interviews were held with them. The patients’ demographic and interventional information (age, gender, ASA, previous surgical experience [type and number], and planned surgery and anesthesia type) were recorded in these interviews.

### 2.2. Measures and Procedure

Around 1 h before the transfer to the operating room, the Spielberger State–Trait Anxiety Inventory (STAI) and the Amsterdam Preoperative Anxiety and Information Scale (APAIS) were applied to all patients by using the face‐to‐face interview technique. The STAI‐State (STAI‐S) and STAI‐Trait (STAI‐T) was developed by Spielberger in 1983, and the inventory was adapted to Turkish by Oner et al. [10], including a validity and reliability study. STAI‐S includes statements that identify how a person feels at the moment, while STAI‐S statements identify how a person feels in general. Each scale has 20 items. While the responses on the STAI‐S scale express 1 = never, 2 = somewhat, 3 = a lot, and 4 = completely, on the STAI‐T scale, the statements are scored as 1 = almost never, 2 = sometimes, 3 = most of the time, and 4 = almost always. The total score obtained from each scale is between 20 and 80. Those with a total score of 20–37 are classified as “low anxiety,” those with a score of 38–44 as “moderate anxiety,” and those with a score of 45–80 as “high anxiety” [11]. Another scale used in the evaluation of preoperative anxiety was the APAIS, which was developed by the Moermann group in the Netherlands in 1996 [12]. In this test, the source of concern is divided into three: anxiety about anesthesia (APAIS‐A), anxiety caused by a lack of information (APAIS‐B), and anxiety about surgery (APAIS‐C). The scale includes 6 statements aimed at these three sources to evaluate anxiety. To ensure the objectivity of the scale, each statement is given a numerical value based on a 5‐point Likert‐type scale according to intensity; these values range from 1 to 5: 1 = none, 2 = little, 3 = moderate, 4 = a lot, and 5 = always. Anesthesia anxiety is calculated by summing the scores given to questions 1 and 2, surgical anxiety questions 4 and 5, and the total anxiety score is the sum of the two scores. The statements expressing the desire to obtain information about anesthesia and surgery are included in questions 3 and 6. The lowest score to be obtained from the scale is 6, and the highest score is 30. It was used by Aykent et al. [13] for the first time in our country.

Intraoperative period routine oral and maxillofacial surgery and anesthesia protocol were applied to the patients whose preoperative anxiety levels were recorded. As a routine procedure, 2 mg/kg propofol, 1 mcg/kg fentanyl, and 0.05 mg/kg rocuronium were used for anesthesia induction; 2% sevoflurane in 50% O_2_ and 50% N_2_O mixture was used for anesthesia maintenance. Paracetamol 10 mg/kg IV was administered as a routine analgesia protocol, and ondansetron 10 mg/kg IV was administered as a routine antiemetic protocol 15 min before the end of the surgery. Rescue analgesia was determined as dexketoprofen 10 mg IV. Surgical and anesthesia procedures, as well as the control procedures for pain, nausea, and vomiting, were applied in the same way for all patients, and all procedures were performed under general anesthesia to ensure standardization of perioperative conditions and to minimize variability related to anesthesia type.

After the planned number of patients was reached, patients were divided into two groups according to the type of surgery: the minor surgery group, which included patients who underwent unilateral impacted maxillary and mandibular tooth extraction, and the major surgery group, which included patients who underwent orthognathic surgery, benign tumor resection, cystectomy, and open reduction of fractures. Cystectomy and benign tumor resection procedures were classified as major surgery due to the extent of surgical intervention, particularly in cases involving large lesions located in anatomically complex regions such as the posterior mandible, where surgical access is more challenging and associated with greater tissue manipulation. The preoperative anxiety scores of the groups obtained from the anxiety scales were compared. In addition, the relationship between these preoperative anxiety scores and postoperative pain and recovery duration was examined.

### 2.3. Data Analysis

Statistical analyses were performed using IBM‐SPSS Statistics software (Version 27), and a *p*‐value of <0.05 was considered statistically significant. Sample size calculation was done based on the anxiety score from a study by Tunc et al. [9]. The required minimum sample size was calculated using a specialized software package for power analysis (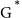
Power Version 3.1.9.4, Heinrich Heine University, Düsseldorf) with 95% power, an effect size of 0.758, and *α* = 0.05. The minimum number of patients required in each group was 47, resulting in a total sample size of 94. We increased the total sample size to 120 to account for the attrition rate. The distribution of continuous variables was primarily assessed using the Shapiro–Wilk test for normality. The Kolmogorov–Smirnov test was applied as a supplementary test, particularly for larger sample sizes. Continuous variables with a normal distribution (*p*  > 0.05 on the Shapiro–Wilk test) were presented as mean ± standard deviation (SD) and compared between two groups using the independent samples *t*‐test. Continuous variables that did not meet the normality assumption (*p*  < 0.05 on the Shapiro–Wilk test) were presented as median and interquartile range (25th–75th percentiles) and compared using the Mann–Whitney *U* test. Categorical variables were expressed as frequencies and percentages. Group comparisons for categorical variables were performed using the Chi‐square test, provided that the expected frequency in each cell was ≥5. If any expected cell frequency was less than 5, Fisher’s exact test was used instead. All statistical tests were two‐tailed, and *p*‐values less than 0.05 were considered statistically significant.

## 3. Results

The study included a total of 120 patients, 58 women and 62 men. The demographic and perioperative data of the patients and the distribution of surgeries performed are presented in Table 1. The most frequent major surgery performed was cystectomy with 46.67%. The most frequently performed minor surgery was impacted mandibular tooth extraction with 63.3%. No significant difference was found between the groups in terms of age, weight, and gender distribution. The duration of surgery was longer in the major surgery group; however, this difference did not reach statistical significance.

**Table 1:** Demographic and perioperative data of patients and distribution according to surgical operations.

Variables	Group min (*n*:60)	Group Mj (*n*:60)	*p*‐Value
Age (years)	32.18 ± 12.42	37.45 ± 16.05	0.047 ^∗^
Weights (kg)	74.16 ± 21.15	71.26 ± 15.36	0.392
Sex (F/M) (*n*) (%)	33/27(55.0/45)	25/35 (41.7/58.3)	0.144
Duration of surgery (dk) (%)	72.53 ± 9.47	117.55 ± 12.21	0.065
Previous surgical experience (*n*) (%) No/minor/major	28/17/15 (46.7/28.3/25)	22/20/18 (36.7/33.3/30)	0.539
Minor surgery (*n*) (%)			
Maxillary impacted third molar extraction	22 (36.7)	—	—
Mandibular impacted third molar extraction	38 (63.3)	—	—
Major surgery (*n*) (%)			
Orthognathic surgery	—	19 (31.67)	—
Cystectomy and benign tumor resection	—	28 (46.67)	—
Maxillofacial traumas	—	13 (21.66)	—

*Note:* Values in number (%), data are presented as mean ± SD.

Abbreviations: Group min, group minor; Group Mj, group major.

^∗^Significant differences are at *p*  < 0.05.

Preoperative anxiety scores obtained from the scales are presented in Table 2. There was a statistically significant difference between the two groups in terms of STAI‐S and STAI‐T anxiety scores and identified anxiety levels. Patients undergoing minor surgery had statistically significantly higher STAI‐S and STAI‐T scores compared to patients undergoing major surgery. With Regarding APAIS‐B levels, patients in the minor surgery group had statistically significantly higher APAIS‐B levels compared to the major surgery group (83.3% vs. 66.7%, respectively; *p*  < 0.05).

**Table 2:** Preoperative anxiety levels of groups.

Variables	Group min (*n*:60)	Group Mj (*n*:60)	*p*‐Value
STAI‐S	49.15 ± 12.82	43.26 ± 13.70	0.017 ^∗^
STAI‐S *n* (%) Level 0/1/2	12/6/42 (20/10/70)	26/5/29 (43.3/8.3/48.3)	0.022 ^∗^
STAI‐T	45.65 ± 10.97	39.90 ± 7.48	0.001 ^∗^
STAI‐T *n* (%) Level 0/1/2	13/9/38 21.7/15.0/63.3	19/21/20 31.7/35.0/33.3	0.003 ^∗^
APAIS T	19.53 ± 6.19	18.50 ± 6.67	0.381
APAIS T level +/−	10/50 (16.7/83.3)	9/51 (15/85)	0.407
APAIS A	5.90 ± 2.18	5.15 ± 2.46	0.081
APAIS B	6.78 ± 2.29	6.71 ± 2.51	0.880
APAIS B level +/−	10/50 (16.7/83.3)	20/40 (33.3/66.7)	0.035 ^∗^
APAIS C	6.98 ± 2.52	6.58 ± 2.40	0.375

*Note:* Values in number (%), data are presented as mean ± SD. Anxiety about anesthesia (APAIS‐A), anxiety caused by lack of information (APAIS‐B), and anxiety about surgery (APAIS‐C). Level 0/1/2: mild/moderate/high, +/−: high/low. STAI‐S and STAI‐T, Spielberger State‐Trait Anxiety Inventory‐State and Spielberger State–Trait Anxiety Inventory‐Trait.

Abbreviations: APAIS, Amsterdam Preoperative Anxiety and Information Scale; Group min, group minor; Group Mj, group major.

^∗^Significant differences are at *p*  < 0.05.

The comparison of postoperative discomfort and recovery durations of the major surgery group and the minor surgery group is presented in Table 3. The pain scores of the patients in the major surgery group at hour 1 were significantly higher than those of the patients in the minor surgery group. However, no significant difference was found between the pain scores at hour 4. Although the incidence of postoperative nausea and vomiting (PONV) did not differ significantly between groups, the number of vomiting was higher in the major surgery group. The postoperative recovery durations after general anesthesia were longer in the major surgery group compared to the minor surgery group, and the difference was statistically significant.

**Table 3:** Comparison of postoperative discomforts and recovery outcomes of the groups (mean ± SD).

Variables	Group min (*n*:60)	Group Mj (*n*:60)	*p*‐Value
VAS 1 (1 h) (mean ± SD)	1.56 ± 2.27	3.43 ± 2.97	*p* < 0.001 ^∗^
VAS 4 (4 h) (mean ± SD)	0.46 ± 0.87	0.78 ± 1.04	0.074
Nausea +/− *n* (%)	20/40 (33.3/66.7)	26/34 (43.3/56.7)	0.260
Number of nausea	0.40 ± 0.61	0.56 ± 0.85	0.222
Vomiting +/− *n* (%)	9/51 (15/85)	17/43 (28.3/71.7)	0.076
Number of vomiting	0.15 ± 0.36	0.36 ± 0.71	0.038 ^∗^
Recovery time	6.51 ± 2.31	10.40 ± 4.22	*p* < 0.001 ^∗^

*Note:* Data are presented as mean ± SD.

Abbreviations: Group min, group minor; Group Mj, group major; VAS, visual analog scale.

^∗^Significant differences are at *p*  < 0.05.

## 4. Discussion

In oral and maxillofacial surgery, most patients have a certain level of anxiety in the preoperative period [3]. The complexity of surgeries is an effective factor in anxiety scores [14]. All procedures can be classified as minor or major surgery depending on the location of the surgery and the tissues involved. While surgeries such as impacted third molar tooth extraction, preprosthetic surgery, and endodontic surgery are categorized as minor surgical operations, orthognathic surgery, maxillofacial trauma management, treatment of cysts and tumors in the maxillofacial region, and TME surgery are classified as major surgical operations [15, 16]. Major and minor procedures may lead to different levels of anxiety scores in patients. This study aimed to determine whether preoperative anxiety, as measured by STAI‐S and STAI‐T, varies by the type of surgery performed under general anesthesia (major vs. minor), with the null hypothesis that there is no difference between the two surgical types and the alternative hypothesis that a difference exists. The mean STAI‐S and STAI‐T anxiety scores were 43.26 ± 13.70 and 39.90 ± 7.48 in the group undergoing major surgery, respectively, while the mean STAI‐S and STAI‐T anxiety scores were 49.15 ± 12.82 and 45.65 ± 10.97 in the group undergoing minor surgery. These results challenge the traditional expectation that major procedures lead to greater anxiety and are consistent with findings from some previous studies suggesting higher information needs or increased anxiety in different procedures. For example, Muglali et al. [17] reported mean STAI‐S/STAI‐T scores of 42.17 ± 9.82 and 42.33 ± 7.33 for minor procedures, and Bozkurt et al. [18] reported STAI‐S of 37.3 ± 8.5 and STAI‐T of 38.6 ± 6.7 in orthognathic patients. Firmeza et al. [19] reported higher anxiety in head and neck pathologies (mean STAI‐S ~41.9). Reasons for these different anxiety scores may include different information needs, expectations regarding postoperative pain and recovery, and differences in the prevalence of surgical anxiety. Therefore, it is important to understand that the type of surgery can shape preoperative expectations and the intraoperative experience, and the need for comprehensive, personalized preoperative information and anxiety management strategies should be considered in both minor and major surgeries. In minor surgeries, openly discussing postoperative pain, recovery times, and what to expect can reduce anxiety, while in major surgeries, intensive intraoperative strategies (multimodal analgesia, enhanced antiemetic protocols, and accelerated recovery pathways) can help balance longer recovery times and complications.

To guide interventions to reduce preoperative anxiety, evaluating the determinants of this anxiety is important [14, 20]. Gursoytrak et al. [21] showed that gender, educational level, general health status, prior surgical experience, and especially the type of surgery are risk factors associated with anxiety in oral and maxillofacial patients. In the present study undergoing maxillofacial procedures under general anesthesia, mean preoperative anxiety scores were higher in the minor surgery group than in the major surgery group (STAI‐S: 49.15 ± 12.82 vs. 43.26 ± 13.70; STAI‐T: 45.65 ± 10.97 vs. 39.90 ± 7.48), suggesting that the relationship between surgery type and anxiety is not direct and may reflect higher information needs, pain, and recovery expectations or a greater prevalence of high trait anxiety within the minor surgery group. Although minor procedures such as impacted tooth extractions are typically performed under local anesthesia, these findings raise the possibility that selecting general anesthesia for certain minor operations could influence perioperative management by facilitating intraoperative control and postoperative care. Therefore, evaluating surgery type together with anesthesia indication, alongside other preoperative data, may be effective in guiding targeted preoperative counseling and anxiety‐management strategies across both minor and major maxillofacial procedures.

Many studies have been conducted to determine patients’ desire to be informed and the factors that influence it in the preoperative period [12, 22]. It has been observed that informing patients about what the procedure involves and why it is performed can reduce anxiety levels [23]. In this study, the rate of patients who wanted to be informed was 16.7% in the minor‐surgery group and 33.3% in the major‐surgery group. Farnill et al. [22] reported that the desire to be informed is higher at younger ages, while Kain et al. [24] demonstrated that the desire to be informed does not change with aging. In our sample, the mean age of the minor surgery group was lower than that of the major surgery group. The higher proportion of patients in the major surgery group who wanted information may reflect the presence of relatively more complex operations in this group. Although age can influence the desire to be informed in the preoperative period, as the complexity of operations increases, patients’ desire to be informed may also increase. Additionally, younger patients may express a greater desire to be informed than older patients. Given that the minor surgery group had higher anxiety scores, it could be that anxious individuals are less inclined to seek information. It is also possible that clinicians performing minor procedures, which are more common and involve less manipulation, provide information less readily than for major procedures. Therefore, it may be necessary to provide comprehensive preoperative information to patients undergoing minor surgery in a manner as clear and detailed as that given to patients undergoing major surgery.

Determining the effect of preoperative anxiety on the postoperative course is important for postoperative pain control and patient follow‐up. In a study of impacted third molar extraction, Hosgor et al. [25] demonstrated that patients with higher preoperative anxiety had lower pain tolerance. In a model examining determinants of postoperative pain, Kalkman et al. [26] reported that preoperative anxiety did not affect postoperative pain and identified age, preoperative pain score, type of surgical procedure, and incision size as the primary determinants of postoperative pain. In the present study, although the minor‐surgery group had higher preoperative anxiety scores, their visual analog scale (VAS) pain scores at hour 1 were significantly lower. This may reflect that major surgery patients typically undergo procedures with a wider operative field, including maxillofacial pathology and trauma and orthognathic surgeries, which are longer and involve more extensive bone and muscle structures and tend to yield higher postoperative pain scores. Therefore, while preoperative anxiety is associated with postoperative pain, the type of surgery may be the main determinant of postoperative pain in this study.

PONV, myalgia, and headache can prolong the hospital stay and affect recovery. The incidence of PONV varies widely across procedures and anesthesia regimens, with Alexander et al. [27] reporting PONV in 11.3% of oral‐surgery patients and Silva et al. [28] reporting 40.8% in orthognathic surgery patients. This variation may reflect differences in surgical procedures, anesthesia types, and follow‐up duration. In our study, the number of vomiting in the major surgery group was found to be statistically significant. This may be due to the longer average operative times in the major surgery group. In addition, the recovery duration was found to be longer in the major surgery group than in the minor surgery group. Longer anesthesia duration, driven by longer operations, could contribute to extended recovery. Fleischmann et al. [29] found that surgery type and length of anesthesia were the most significant factors associated with recovery duration. In the present study, recovery duration was significantly longer in patients undergoing major surgery. The finding that minor surgery patients, who had higher preoperative anxiety, showed shorter recovery durations may suggest that surgery type has a greater impact on recovery than preoperative anxiety, though this observation should be interpreted cautiously given potential confounders and sample size. Overall, while preoperative anxiety can influence anesthetic use and hospital stay, the data here indicate that the type of surgery may be the more important determinant of postoperative recovery and complications.

Although the present study has certain limitations, several strengths should also be emphasized when interpreting the findings. While multivariate analysis was not performed to adjust for all potential confounding factors, the study was designed as a prospective comparative observational study, and baseline demographic and perioperative characteristics were comparable between groups. Preoperative anxiety was assessed using validated instruments (STAI and APAIS), and the prospective design with standardized anesthesia and postoperative care protocols minimized procedural variability. Nevertheless, the single‐center nature of the study and the relatively short follow‐up period may limit the generalizability of the findings. In addition, all procedures were performed under general anesthesia, which may influence anxiety levels and limit applicability to procedures commonly performed under local anesthesia. Another consideration is that cystectomy procedures may vary in complexity depending on the lesion size, location, and surgical approach, potentially introducing heterogeneity in the classification of surgical groups. Furthermore, patient‐related factors such as age, comorbidities, prior surgical experience, and individual risk perception may have influenced preoperative anxiety. In particular, the relatively lower mean age in the minor surgery group may have contributed to the higher anxiety scores. Therefore, the observed differences in anxiety may not be solely attributable to the type of surgery.

In addition, the inclusion of patients undergoing different types of procedures, including elective and trauma‐related surgeries, may have introduced heterogeneity in the clinical context and baseline psychological conditions. Patient expectations and prior perceptions regarding the planned surgical procedure may also represent important confounding factors that were not directly assessed in this study. Furthermore, postoperative pain and recovery outcomes may vary depending on the type and extent of surgical procedures, which may have contributed to the variability in postoperative results. These factors should be considered when interpreting the findings, and the results should be evaluated with caution.

## 5. Conclusion

In this prospective study of 120 patients undergoing oral and maxillofacial surgery under general anesthesia, preoperative anxiety levels differed according to the type of surgery. Higher STAI‐S and STAI‐T scores, as well as higher APAIS scores, were observed in the minor surgery group compared to the major surgery group. Although preoperative anxiety was higher in minor procedures, patients in the major surgery group experienced greater early postoperative pain at 1 h, a higher frequency of vomiting within the first 6 h, and longer recovery times. These findings suggest that the extent of the surgical procedure may be an important factor influencing early postoperative outcomes. However, it should be noted that preoperative anxiety and postoperative outcomes are likely influenced by multiple factors, including patient‐related characteristics such as age, general health status, and individual perception of surgical risk. Clinically, these findings indicate that preoperative and postoperative planning may benefit from a procedure‐specific approach. While improving information provision and anxiety management for minor surgeries, greater emphasis may be placed on multimodal analgesia, antiemetic strategies, and enhanced recovery protocols in major surgical procedures. Future research should further explore the interaction between anxiety, patient characteristics, and surgical factors, as well as the impact of targeted anxiety‐reduction interventions on postoperative outcomes.

## Author Contributions

Zeynep Busra Gur contributed to conceptualization, investigation, formal analysis, writing – original draft, and writing – review and editing. Ozlem Kocaturk contributed to investigation, formal analysis, writing – original draft, and writing – review and editing. Burcu Gursoytrak contributed to review and editing.

## Funding

This research received no external funding.

## Ethics Statement

Ethical approval for this study was obtained from the Ethics Committee of Aydın Adnan Menderes University (Approval Number AADUDHF2023/28, Date: 15/03/2023). All procedures were conducted in accordance with the Declaration of Helsinki. Informed consent was obtained from all participants.

## Conflicts of Interest

The authors declare no conflicts of interest.

## Data Availability

The data that support the findings of this study are available upon request from the corresponding author. The data are not publicly available due to privacy or ethical restrictions.
